# Nutritional quality and adulterants of cow raw milk, pasteurized and cottage cheese collected along value chain from three regions of Ethiopia

**DOI:** 10.1016/j.heliyon.2023.e15922

**Published:** 2023-05-03

**Authors:** Haftom Zebib, Dawit Abate, Ashagrie Zewdu Woldegiorgis

**Affiliations:** a Center for Food Science and Nutrition, College of Natural Sciences, Addis Ababa University, Addis Ababa P.O. Box 1176, Ethiopia; b Livestock and Fishery Core Process, Tigray Agricultural Research Institute, Mekelle P.O. Box 492, Ethiopia; c Department of Biology, College of Natural Sciences, Addis Ababa University, Addis Ababa P.O. Box 1176, Ethiopia

**Keywords:** Added water, Adulterants, Minerals, Nutritional, Value chains, Raw milk, Pasteurized milk, Cottage cheese

## Abstract

Milk is a nutritionally rich food for humans. However, fulfilling the quality of milk is a major concern for milk factories, nutrient requirements, and public health. The objective of this research was to assess the composition of raw and pasteurized milk and cheese, evaluate change in milk and cheese composition along the value chain, and identify adulteration of milk. A total of 160 composite samples were determined using lactoscan and conventional approved methods along value chain. Results indicate that there were significant (*p* < 0.05) changes of in milk composition along the value chain in the study regions. The range values were; total solid (8.41–11.7%), protein (2.25–3.06%), fat (2.16–3.17%), lactose (3.33–4.76%), ash (0.52–0.73%), P (62.7–84.2 mg/100 g) and Ca (78.2–109 mg/100 g) of liquid milk were obtained in all regions. Liquid milk was found to be adulterated by water along the value chains in all regions (ranged from 0 to 24.8%). Formalin (4 samples) and starch (1 sample) were detected at farmer's and collectors' respectively. In all regions, there was no significant (*p* > 0.05) difference in cheese nutritional quality between farmers and retailers. The grand mean for moisture, protein, fat, total ash, Ca, P and pH values were 77.1%, 17.1%, 1.42%, 1.18%, 37.8 mg/100 g, 88.2 mg/100 g and 3.7 respectively. Comparison of liquid products with the Compulsory Ethiopian Standard (CES) indicates that 80.2% for fat, protein, and SNF in raw and pasteurized milk were below the CES. In conclusion, liquid milk had poor nutritional composition and varied along the value chain in the study regions. Moreover, there is milk fraud where all dairy value chain add water into milk and milk consumers are consuming lower nutrients and paying for substandard liquid milk. Therefore, training should be provided to all value chain to improve the quality of milk products and quantification of formalin and other adulterants need to be further studied.

## 1 Introduction

Livestock and dairy production play an important role in supporting livelihoods, nutrition, and economies in Ethiopia. It is believed to have the largest livestock population in Africa (about 65 million cattle, 51 million goat, 40 million sheep, 49 million chicken and 8 million camels) [1]. The livestock sector holds a share of 15–17% of gross domestic product (GDP), 35–49% of agricultural GDP and 37–87% of household incomes [2]. The distribution of different milk-producing livestock species differs from one region to another [3]. Oromia produces 42.3%, SNNP 22.7%, Amhara 20.5%, and Tigray 6.8% of the total cow milk production volume (3,284, 452, 075 liters) in Ethiopia [4]. Milk products are consumed in rural, urban, and peri-urban areas by different age groups in different regions. For the period 2003 to 2012, Ethiopia's per capita milk consumption ranged from 32.8 to 36.5 L per head/year [5], which is lower than in Kenya (between 50 and 150 L per capita per year [6,7].

Dairy chains link the actors and activities involved in delivering milk products to the final consumer which involves production, processing, packaging, storage, transport and marketing. In Ethiopia, dairy products are marketed to customers and milk factories via both formal and informal dairy marketing systems. About 95% of the country's milk is sold informally, with farmers selling to neighbors, cooperatives, and retailers [8]. Raw milk is a key ingredient in the production of pasteurized milk, cottage cheese, yoghurt, and whey in the industry [9,10].

Milk is a highly nutritious and perfect natural food for humans and animals. The important role of cow's milk in the human diet is as a supplier of energy, water, protein, fat, lactose, and vitamins. They are also good sources of major minerals, particularly calcium, phosphorus, magnesium, potassium, and trace elements such as zinc. Milk composition is influenced by bovine breed, age, health status, stage of lactation, diet; the intensity of management; milking interval; and ambient environmental temperature and seasonality [11,12]. Other factors contributing to milk profile differences include feeding patterns, geographical locations, analytical techniques used, and number of samples [13].

Milk adulteration is the alteration of the natural composition of milk by altering more of its components and the addition of water by a value chain for economic gain. Adulteration interferes with the compositional, processing quality of milk and reduce the nutritional value of milk [14,15]. Other substances, such as formaldehyde, hydrogen peroxide, hypochlorite, dichromate, and salicylic acid, have been added to increase the product shelf life in different countries [16,17]. Furthermore, the adding of table sugar, soap, starch, salt, urea, detergent, skim milk powder, and alcohol is a practice that can alter the quality standard of processed products and adversely affect human organs [18]. Generally, some substances are intentional added into milk products and some others are due to contamination or poor practices.

In Ethiopia, raw milk adulteration by water addition and the compositional quality of liquid milk in the Addis Ababa retail market [19] and cottage cheese [20] were reported. As far as we know, there is no information on variation and changes of in nutritional quality and detection of other adulterants in the context of a value chain with a composite representative large sample size in major potential producing regions in the country. The objectives of this paper were(i) to assess the composition of raw and pasteurized milk and cheese produced in 3 regions of Ethiopia, (ii) to evaluate change in milk and cheese composition along the value chain, and (iii) identify adulteration of milk.

## 2 Materials and methods

### 2.1 Study area

**Fig. 1:**
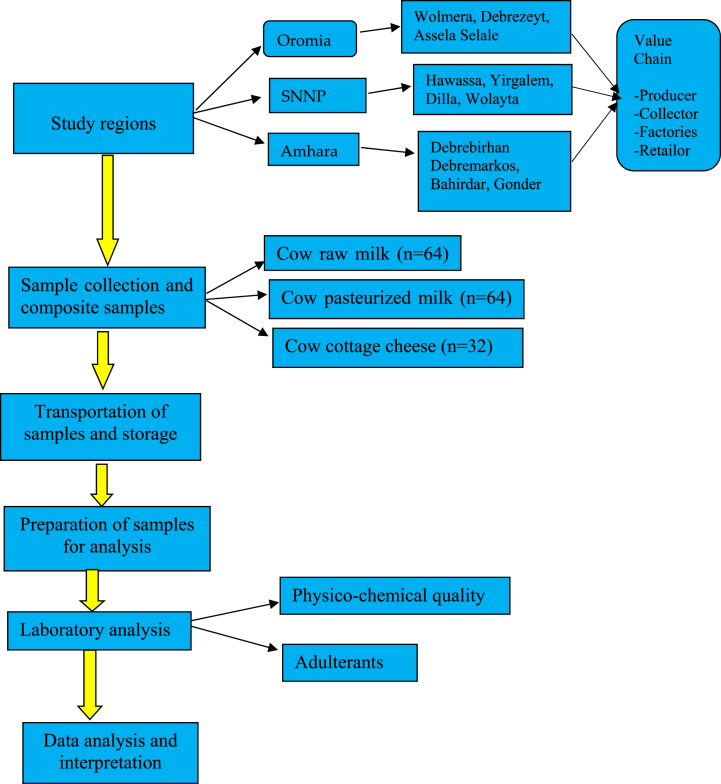
Flow diagram of research methodology.

According to the Central Statistical Agency (CSA), sample collecting sites were selected based on locations where milk production is high [4]. The CSA of Ethiopia is responsible for the statistical data generation related to the socio-economic condition of the country. The study regions were urban and peri-urban areas of Oromia's, SNNP (Southern Nations,Nationalities and People) including the current Sidama and Amhara regions. For value chains such as farmers, collectors, milk factories, and retailers were participated in the study regions. In each region, four district areas were selected. Selale, Asela, Debrezeyt/Bishoftu, and Wolmera in Oromia; Wollayta, Dilla, Yirgalem and Hawassa in SNNP; and Gondar, Bahirdar, Debremerkos and Debrebirhan in Amhara region. Fig. 1. Shows summary of research methodology.

### 2.2 Collection of samples, transportation and storage

In the dry season, all primary samples were collected in the Oromia area from January to April 2019, and in the SNNP and Amhara regions from December 2019 to March 2020. Because of the COVID 19 pandemic, the sampling period was interrupted. Raw milk samples were collected from farmers and collectors using sterilized equipment, sampling apparatus, and containers in accordance with [21]. Pasteurized milk samples processed at a high temperature short time (72 °C, 15 s) were collected from milk factories and retailers, whereas raw milk samples were collected from farmers and collectors. Primary raw milk samples (about 200 ml) were taken in sterile screw-capped clean plastic bottles (250 ml capacity). Pasteurized milk (500 ml capacity) was purchased before its expiration date from milk factories and supermarkets/retail shops. To ensure that various batches of pasteurized milk from factories and raw milk samples from collectors were collected, different day intervals were used for collecting. Ethiopian cottage cheese (about 200 g) was collected in a zipper polyethylene plastic bag from farmers and the local retail market. Within 48 h of collection, all samples were transported in a portable refrigerator (Dometic, CFX-50 W, China) at 4 °C to the Addis Ababa University-Center for Food Science and Nutrition laboratory. Samples were immediately analyzed for compositional quality determinations and then held at −20 °C until mineral determinations were made.

### 2.3 Sampling and sample size

For the selection of value chains, farmers and retailers were chosen using simple random technique, whereas collectors (4 in each study district within the study regions) and milk factories (Oromia = 4, SNNP = 1 and Amhara = 2) were chosen purposefully. Collectors and factories were small in size and all were considered for sampling. For evaluation, 160 composite samples of raw milk, normally pasteurized milk, and cottage cheese were used. According to Ref. [22], this was done by combining five primary samples and taking about 50 g per sample to obtain one representative composite sample along the value chain (farmers, collectors, milk factories, and retailers) in each region. Table 1 shows composite sample size in each region along the value chain and total size of value chain participants considered in the study.

**Table 1:** Composite sample size of milk products along the value chain.

Value chain	Product type	Study regions			Total
		Oromia	SNNP	Amhara	
Farmers (n = 240)	Raw milk	16	8	8	32
	Cottage cheese	8	4	4	16
Collectors (n = 12)	Raw milk	16	8	8	32
Factories (n = 7)	Pasteurized milk	16	8	8	32
Retailers (n = 240)	Pasteurized	16	8	8	32
	Cottage cheese	8	4	4	16
Total		80	40	40	160

### 2.4 Production procedure for local cottage cheese

Traditional cottage cheese is fresh cheese with a lower pH value. To prepare this, first raw milk is fermented under ambient temperature for 2–3 days to make traditional ergo (traditional yoghurt) [3]. Then it's processed, which differs from soft cheese made in other countries, by churning the ergo and removing the cream, then gently heating the butter milk and draining off the whey. This is made without addition of rennet and coagulants, which is practiced in different countries.

### 2.5 Preparation of milk and cheese samples

The sample was prepared according to the approved AOAC [23] method 925.21. Deep freeze samples were brought to a temperature of 20 °C. When homogenous fat dispersion in milk was not achieved, the sample was slowly warmed in a water bath (Biobase, WB-82, China) to 37 °C, then gently mixed by repeated inversion and cooled to 20 °C.

### 2.6 Compositional quality and adulterants of raw and pasteurized milk

An ultra-sonic spectroscopy device (Milcotronic, Lactoscan SP, Bulgaria) was used to determine the major chemical composition of raw and pasteurized milk samples, including fat, protein, lactose, solids-not-fat (SNF), pH, and extent of added water. The analysis was carried out in accordance with the manufacturer's instructions. The pH of cottage cheese was determined by a pH meter (HFCC, PHS-3DW, China). Before analyzing the sample, the pH meter was calibrated using 4.0 and 7.0 buffer solutions. Cottage cheese (about 2 g) was homogenized (2:20; w: v) in distilled water. The pH was then determined by submerging the electrodes in liquid milk until a stable reading was obtained.

Formalin was analyzed in milk by the method [24]. About 5 ml of milk was taken in a test tube. 1 ml of 10% ferric chloride solution was taken in a 500 ml volumetric flask and made up the volume using concentrated hydrochloric acid. 5 ml of this solution was added to the sample in the test tube. Thereafter, it was kept in the tube in a boiling water bath for about 3–4 min. The appearance of a brownish pink color will indicate the presence of formalin. Positive (3% formalin) and negative controls were used as comparisons. Starch was analyzed by the method described by Ref. [24]. A 3 ml sample was taken in a test tube. After boiling, it was thoroughly mixed and cooled at room temperature. Afterwards, 1 drop of 1% iodine solution was added. The appearance of blue indicates the presence of starch. The detection range for formalin and starch was 2%.

### 2.7 Nutritional composition of milk and cottage cheese

The nutritional parameters of all milk products were analyzed using approved methods.

#### 2.7.1 Determination of total solids and moisture in milk and cheese

Total solid of the milk was determined according to Ref. [23] method 990.20 and the moisture content of cheese was determined using [23] method 926.08. Briefly, about 5 g of sample was taken and transferred to a pre-weighed and dried crucible at 100 °C for 1 h. The samples in the crucibles were gently heated at 45–50 °C on a hot plate stirrer (Biocote, UC 152, England) to evaporate the majority of water and avoid spattering. Then the partially dried samples were dried in a forced air oven (Genlab, OV/125/SS/F/D/G/A, UK) at 100 °C for 4 h. Finally, the dried samples were taken out of the oven and placed in a desiccator (Findel, CNF-Simax, England) to cool to room temperature for about 30 min. The samples were weighed again and total solids were calculated by the weight differences according to equation [1].

[1] $$Total\;solids\;\left(\%\right)=\frac{\left(M_{3}-M_{1}\right)}{\left(M_{2}-M_{1}\right)}\times 100$$

Where, M_1_ = Weight of crucible (g).

M_2_ = Weight of sample and crucible before drying (g)
M_3_ = Weight of sample and crucible after drying (g)

#### 2.7.2 Determination of total ash in raw and pasteurized milk and in cheese

The ash content of liquid milk and cottage cheese was determined according to Ref. [23] method 945.46. and 935.42 respectively. In brief, the dried milk used for the determination of total solids/moisture was ignited in a Muffle Furnace (Carbolite, CFS 1200, England) at ≤550 °C until ash was carbon free. Then the samples were transferred to the desiccator to cool down for about 30 min. The dish containing the sample was then re-weighed after the dish was completely cooled. The total ash percent of the sample was calculated by weight differences using equation [2]:

[2] $$Total\;ash=\frac{\left(M_{3}-M_{1}\right)}{\left(M_{2}-M_{1}\right)}\times 100$$

Where, M_1_ = Weight of crucible (g).

M_2_ = Crucible + sample weight (g)
M_3_ = Final weight of sample + crucible (g)

#### 2.7.3 Determination of crude protein in cottage cheese

This was determined according to method 2001.14 using Kjeldahl method. 1 g of sample was poured into a digestion flask containing catalyst, 1 g of a 10:1 mixture of Na_2_SO_4_ and anhydrous CuSO_4_, and 5 ml of concentrated H_2_SO_4_. Digestion was carried out using digestion apparatus. The Kjeldahl flask was heated to temperature of 350 °C on digestion apparatus using a heater, and digestion was continued until the digest was clear. The acid digest was allowed to cool to room temperature. Distillation was performed by adding 30 ml of distilled water, 25 ml 40% NaOH to a Kjeldahl flask and connecting it to a nitrogen analyzer automatically distillation device (Hua Ye, KDN-102 F, China), whose outlet tube was immersed in 25 ml of 4% boric acid solution. The distillate (150 ml) was collected and titrated with standard acid (0.1 N HCl). The volume of HCl consumed was taken from the burette reading, and then the %N protein was calculated by multiplying the conversion factor (6.38) in the equation described below.

[3] $$N=\left(\frac{V_{HCl}\;x\;N_{HCl}x14}{M}\right)X\;100$$

Where, N = Nitrogen of sample (%)

V_HCl_ = Volume of HCl used in the titration (l)
N_HCl_= Normality of HCl (about 0.1)
14 = Molecular mass of N
M = Sample mass (g)

#### 2.7.4 Determination of fat in cottage cheese

This was carried out using the gravimetric method according to Ref. [25] method 1735. A sample (about 3 g) was weighed and added directly to the fat extraction flask (Siphon). Then 10 ml of dilute hydrochloric acid (1.13 g/ml) was added so as to wash out the test portion fat extraction flask, and mixed well. Afterwards, it was heated gently on a hot plate until all the particles were entirely dissolved, and then allowed the flask to stand for 20–30 min on the hot plate and cooled under running water. Then 10 ml of ethanol (97%) was added and mixed gently but thoroughly by allowing the contents of the flask to flow backwards and forwards between the two bulbs. Next, 5 ml of diethyl ether was added to each flask and the fat-extraction flask was closed with a cork saturated with a stopper wetted with water. The flask was shaken vigorously for 1 min, but not so vigorously as to cause the formation of a persistent emulsion. The stopper was removed carefully and the neck of the flask was rinsed with a little mixed solvent (mixing equal volumes of diethyl ether and light petroleum ether) using the wash bottle so that the rinsing ran into the fat-extraction flask. Afterwards, 25 ml of the light petroleum was added and the fat-extraction flask was closed with the rewetted cork or rewetted stopper (by dipping in water). Then the flask was gently shaken for 30 s and allowed the closed tube to stand in the rack for at least 30 min until the supernatant layer was clear and distinctly separated from the aqueous layer. The cork was removed carefully and the neck of the tube rinsed with a little mixed solvent. The supernatant layer out of the tube is carefully transferred into the beaker to avoid transfer of any of the aqueous layer. The outlet of the fitting was rinsed with a little mixed solvent, collecting the rinse in the beaker. A second extraction was carried out by adding 15 ml of diethyl ether and 15 ml of light petroleum. The inside of the neck of the flask was rinsed with a little mixed solvent. The fat-collecting vessel was heated in the drying oven set at 102 °C for 1 h Until the fat was clear and weighed. A blank test was carried out simultaneously with the determination. The mass fraction of fat (w) of the sample was calculated using the following equation.

[4] $$W=\frac{\left(M_{1}-M_{2}\right)-\left(M_{3}-M_{4}\right)}{M_{o}}$$

Where, M_0_ = mass of the test portion (g).

M_1_ = mass of the fat collecting vessel (g)
M_2_ = mass of the prepared fat collecting vessel (g)
M_3_ = mass of the fat collecting vessel used in the blank test and any extracted matter in it (g)
M_4_ = mass of the fat collecting vessel used in the blank test (g)

#### 2.7.5 Determination of calcium in raw and pasteurized milk

This was determined using the titrimetric method according to Ref. [26] method 12,081. Samples (about 20 ml) were pipetted into a volumetric flask (capacity 50 ml). Then trichloroacetic acid-I (200 g/l) was added until 50 ml was added gradually with vigorous shaking for a few seconds and allowed to stand for 30 min. The mixtures were filtered through asheless filter paper (125 mm). For precipitation of calcium as oxalate and separation of the oxalate, a 5 ml clear filtrate sample was taken and added to a centrifuge tube followed by 5 ml trichloro acetic acid solution-II (120 g/l), 2 ml ammonium oxalate saturated solution, 2 drops of methyl red solution (0.05%), and 2 ml acetic acid solution by mixing and swirling. Then ammonia solution (50%) was added drop by drop until the color became pale yellow. Then a few drops of acetic acid solution (20%) were added and allowed to stand for 4 h at room temperature until a pink coloration appeared. The contents of the centrifuge tube were diluted with distilled water to 20 ml. The test tube was centrifuged using a centrifuge device (Wincom, 800, China) at 1400 *g* for 10 min. Then the clear supernatant liquid was removed from the centrifuge tube with the help of a transfer pipette. The walls of the centrifuge tube were rinsed with 5 ml of ammonia solution II (2%) while taking care not to disturb the deposit of calcium oxalate. The test tube was centrifuged at 1400 g for 5 min. Then the clear supernatant liquid was removed from the centrifuge test tube by a transfer pipette. This washing operation was repeated twice. For titration, 2 ml of sulfuric acid (20%) and 5 ml of distilled water were added to the calcium oxalate deposit. Then the test tubes were placed in the boiling water bath to dissolve the calcium oxalate deposit completely. Afterwards, the dissolved calcium oxalate was titrated with the potassium permanganate solution (0.004 M) until a pink color persisted. During the titration, the temperature of the solution stayed above 60 °C. The volumes (ml) of potassium permanganate solution used for sample titration and blank samples were recorded. A blank test was carried out in parallel with the determination by using 20 ml of water instead of the test portion. The Ca content was calculated and expressed as a percentage mass fraction, using the following equation [5]:

[5] $$Ca=0.4\;\left(V-V_{O}\right)*1000\frac{F}{M}$$

Where; V = the volume of potassium permanganate solution used for the test portion(ml)

V_0_ = is the volume of potassium permanganate solution used for the blank test (ml)
M = the mass, in grams, of the test portion;
F = the correction factor

#### 2.7.6 Determination of calcium in cottage cheese

An atomic absorption spectrophotometer (ZEEnit, 700 P, Germany) was used for Ca analysis in cheese [23] Method 991.25. A sample (about 1 g) was weighed into an ashing vessel (that had been pre-ignited at 550 °C and cooled in a desiccator) and dried in an oven at 100 °C for 1 h. The sample was ashed in the muffle furnace at 525 °C until ashing was completed. Then, the ash was dissolved in 1 ml of HNO_3_, transferred to a 250 ml volumetric flask, and diluted to volume (sample solution). The solution was boiled and evaporated nearly to dryness and was filtered through coarse porosity filter paper into a 100 ml volumetric flask. A concentrated stock solution of Ca was prepared by adding analytical grade 1.25 g CaCO_3,_ 30 ml of 3 M HCl, and then it was diluted to 1000 ml. A series of standard solutions (0, 5, 15, 20, 25 & 30 ml in a 100 ml volumetric flask) were prepared from aliquots of dilute stock solution of Ca (20 mg/L). Finally, the absorbance of the sample was measured at 422.7 nm by taking 10 ml of sample solution, adding 10 ml of La stock solution (dissolving 11.7 g LaO_3_ in 25 ml of HNO_3_ and diluting to 1000 ml) and diluting to a 100 ml flask. The calcium content was calculated with the following equation [6].

[6] $$Ca\;\left(mg/100g\right)=\frac{Z\times 2500}{W/V}$$

Where: Z = concentration of the sample from plot of absorption in μg/ml.

W = sample weight (g)
V = volume of sample taken for assay, ml

#### 2.7.7 Determination of phosphorus in raw and pasteurized milk

This was determined according to Ref. [23] method 986.24 by molybdovanadate reagent using a UV–Vis/NIR spectrophotometer (PerkinElmer, Lambda 950, UK). The Molybdovanadate reagent was prepared by dissolving 20 g of ammonium molybdate in 200 ml of hot water, then dissolving 1 g of ammonium metavanadate in 125 ml of hot water, to which when cooled, was added 140 ml of concentrated nitric acid. The cooled vanadate and molybdate solutions were then combined and diluted to 1 L. The prepared molybdovanadate reagent was stored in the dark for further use. For analysis, about 2 g of dried milk was ashtray using a muffle furnace at 600 °C for 3–4 h. Then the ash was dissolved in 5 ml of 6 N HCl and several drops of nitric acid and heated to completely dissolve the ash. Afterwards, the digested sample was cooled, filtered, and transferred to a 100 ml volumetric flask by diluting with distilled water. The stock standard solution of P was prepared (2 mg P/ml) by weighing 8.77 g of KH_2_PO_4_ that had been dried at 105 °C for 1 h. From this, a working standard solution (0–1.5 mg of P) was prepared from diluted standard solution (0.1 mg/l P by diluting in 50 ml of stock solution to 1000 ml with distilled water). After adding 20 ml of molybdovanadate reagent and diluting to volume with distilled water, absorbance was measured at 400 nm. The phosphorus content was calculated using equation [7].

[7] $$P\;\left(mg/100\;g\right)=\frac{Y}{W}*DF$$

Where, Y = concentration of the sample from plot of absorption in μg/ml P.

W = Sample weight (g)
DF = Dilution factor

#### 2.7.8 Determination of phosphorus in cottage cheese

This was determined by molybdovanadate reagent using a UV–Vis/NIR spectrophotometer [23]. A sample (about 1 g) was weighed into an ashing vessel (that had been pre-ignited at 550 °C and cooled in a desiccator) and dried in an oven at 100 °C for 1 h. The sample was ashed in the muffle furnace at 525 °C until ashing was completed. Then, the ash was dissolved in 1 ml of HNO_3_, transferred to a 250 ml volumetric flask, and diluted to volume (sample solution). The solution was boiled and evaporated nearly to dryness and was filtered through coarse porosity filter paper into a 100 ml volumetric flask. A concentrated stock solution of phosphorus (300 mg P/l) was prepared by adding analytical grade 1.11 g (NH_4_H_2_PO_4_), 30 ml of 3 M HCl, and then it was diluted to 1000 ml. A series of standard solutions (0, 5, 10, 15, 20, 25, 30 & 35 ml in a 50 ml volumetric flask) were prepared from aliquots of a dilute stock solution of phosphorus (12 mg/L). Finally, the absorbance of the sample was measured at 400 nm by taking 10 ml of sample solution, adding 10 ml of molybdovanadate reagent, and diluting to a 50 ml flask. The P content was calculated from the calibration curve using equation [8].

[8] $$P\;\left(mg/100g\right)=\frac{Y\times 125}{W/V}$$

Where: Y = concentration of the sample from plot of absorption in μg/ml P.

W = sample mass
V = volume of sample taken for assay, ml
Minerals (Ca &P)

### 2.8 Statistical analysis

All values were performed in duplicate analysis and average values were used for data analysis. The data was expressed as mean ± SD. All the data analysis was carried out using Stata/IC 15.1. A linear regression model was used to analyze the data, with the milk and cheese parameters acting as dependent variables and the region and value chains acting as independent variables. Pairwise comparisons of means after linear regression were used to compare the milk and cheese parameters across the dependent variables; significance was determined with DMRT (Duncan's multiple range test) to test mean pairs and was accepted at the probability of *p* *<* 0.05. Microsoft Excel was used to summarize the data.

## 3 Results

### 3.1 Nutritional composition of raw and pasteurized milk along value chain in the regions

The nutritional composition of raw and pasteurized milk along value chain in the study regions are shown Table 2. Total solids in milk are an important indicator of nutritional composition (fats, proteins, lactose and minerals). The mean total solid content of liquid milk was, 8.78–9.82%, 9.29–10.7% and 8.41–11.7% in Oromia, SNNP and Amhara regions respectively. Results showed samples from farmers had significantly (*p* < 0.05) higher total solids than factories and retailers’ samples in the Amhara and SNNP regions. The amount of change from farmers to collectors were decreased by 6.0%, 11.2 and 11.2% for the above regions respectively.

**Table 2:** Nutritional composition of raw and pasteurized milk along value chain in three regions of Ethiopia.

Study regions	Value chain	Product type	N	Total solids (%)	SNF (%)	Fat (%)	Protein (%)	Lactose (%)	Total Ash (%)	Ca (mg/100 g)	P (mg/100 g)	pH
Oromia	Farmers	Raw milk	16	9.82 ± 2.26^abc^	7.10 ± 1.54^bcd^	2.96 ± 0.93^bc^	2.54 ± 0.63^abc^	3.72 ± 0.92^abc^	0.61 ± 0.12^cd^	88.2 ± 6.6^ab^	74 ± 19.7^ab^	6.52 ± 0.39
	Collectors	Raw milk	16	9.29 ± 2.41^ab^	6.88 ± 2.15^abc^	2.53 ± 0.78^ab^	2.53 ± 0.94^abc^	3.62 ± 1.08^abc^	0.58 ± 0.13^abc^	84 ± 8.6^bc^	72 ± 8.8^bc^	6.42 ± 0.34
	Factories	Pasteurized	16	8.78 ± 1.52^a^	6.34 ± 0.99^ab^	2.63 ± 0.51^ab^	2.25 ± 0.43^a^	3.38 ± 0.69^ab^	0.55 ± 0.08^ab^	81.3 ± 5.6^ba^	71.0 ± 10^ab^	6.68 ± 0.32
	Retailers	Pasteurized	16	9.30 ± 1.59^abc^	6.48 ± 1.01^ab^	2.89 ± 0.51^bc^	2.38 ± 0.45^ab^	3.48 ± 0.70^abc^	0.56 ± 0.07^abc^	82.5 ± 5.6b^ab^	70.8 ± 10.8^ab^	6.61 ± 0.43
	**Total/Grand mean**		**64**	**9.30**	**6.70**	**2.75**	**2.43**	**3.55**	**0.58**	**84.0**	**72**	**6.56**
SNNP	Farmers	Raw milk	8	10.7 ± 1.37^bcd^	7.06 ± 0.88^de^	2.71 ± 0.80^abc^	2.93 ± 0.30^bc^	4.38 ± 0.47^de^	0.68 ± 0.07^de^	99.2 ± 9.8^d^	82.6 ± 10.3^c^	6.61 ± 0.26
	Collectors	Raw milk	8	9.46 ± 1.36^abc^	7.21 ± 0.87^bcd^	2.17 ± 0.64^a^	2.66 ± 0.33^abc^	4.05 ± 0.57^cd^	0.62 ± 0.08^bcd^	89.1 ± 15.5^bc^	76.4 ± 13.6^bc^	6.74 ± 0.54
	Factories	Pasteurized	8	9.34 ± 0.53^abc^	7.20 ± 0.43^bcd^	2.16 ± 0.43^a^	2.63 ± 0.15^abc^	3.96 ± 0.24^bcd^	0.60 ± 0.04^abcd^	86.4 ± 3.80^abc^	72.6 ± 12.1^abc^	6.82 ± 0.21
	Retailers	Pasteurized	8	9.29 ± 0.93^abc^	6.77 ± 0.56^abcd^	2.56 ± 0.61^abc^	2.48 ± 0.21^abc^	3.71 ± 0.31^abcd^	0.58 ± 0.05^abc^	84.1 ± 4.78^ab^	67.2 ± 16.75^abc^	6.90 ± 0.13
	**Total/Grand mean**		**32**	**9.69**	**7.06**	**2.40**	**2.67**	**4.03**	**0.62**	**89.7**	**74.7**	**6.76**
Amhara	Farmers	Raw milk	8	11.7 ± 1.45^d^	8.67 ± 0.44e	3.17 ± 0.16^c^	3.06 ± 1.32^c^	4.76 ± 0.24^e^	0.73 ± 0.04^e^	109 ± 12.3^e^	84.2 ± 10.3^c^	6.72 ± 0.10
	Collectors	Raw milk	8	10.7 ± 0.93^cd^	7.92 ± 1.77^cde^	2.90 ± 0.66^bc^	2.82 ± 1.43^abc^	4.35 ± 0.97^de^	0.66 ± 0.13^de^	93.8 ± 13.5^cd^	82.3 ± 7.38^c^	6.52 ± 0.24
	Factories	Pasteurized	8	8.41 ± 1.82^a^	5.90 ± 1.07^a^	2.16 ± 0.39^a^	2.53 ± 0.96^abc^	3.24 ± 0.59^a^	0.52 ± 0.02^a^	79.3 ± 9.88^a^	62.9 ± 5.88^a^	7.18 ± 0.19
	Retailers	Pasteurized	8	8.45 ± 1.01^a^	6.07 ± 0.50^ab^	2.22 ± 0.18^a^	2.40 ± 0.70^abc^	3.33 ± 0.26^ab^	0.52 ± 0.04^a^	78.2 ± 5.54^a^	62.7 ± 5.23^ab^	7.13 ± 0.22
	**Total/Grand mean**		32	**9.82**	**7.14**	**2.61**	**2.70**	**3.92**	**0.61**	**90.1**	**73**	**6.88**

All values were duplicate analysis (n = 2); mean values that do not share common letters, are different for p < 0.05; SNF=Solids Not fat.

The results indicate there were no a significant difference (*p* > 0.05) in raw milk protein content between farmers and collectors in Oromia and between milk factories and retailers in the Amhara region. However, farmers had significant differences (*p* < 0.05) in SNNP region. The percentage of variation for protein was 0.39% (Oromia), 9.22% (SNNP) and 7.84% (Amhara) between farmers and collectors. The highest (3.06%) was recorded in producer in Amhara region while lowest (2.25%) was in samples from factories in Oromia region. In SNNP and Amhara regions, there was no significant differences (*p* > 0.05) between milk factories and retailers in protein content of pasteurized milk.

In all study regions, there were no significant (*p* > 0.05) changes in fat content along the value chain except at the farmers level in Amhara region. Samples collected from the factories and retailers show low fat values in the Amhara region. The fat content ranged from 2.17 to 3.6%. In the Oromia, SNNP, and Amhara regions, fat changes in the value chain show 14.5, 19.9, and 8.52% decreases in collectors from farmers, respectively. The regions mentioned above, from collectors to milk factories, showed reductions of 3.95, 0.46, and 25.5%, respectively.

The lactose content of pasteurized milk collected from factories in the Oromia region was significantly (*p* < 0.05) lower than samples collected from other value chains. In the SNNP and Amhara regions, however, there were no significant changes (*p* > 0.05). In all study areas, the value of lactose was between 3.38 and 3.96%, which is much lower than farmers and collectors (3.55–4.66%). When raw milk reaches collecting sites in Oromia, SNNP, and Amhara regions, the level of lactose in dairy farmer decreases by 2.69, 7.53, and 8.61%, respectively.

Raw milk samples from farmers had significantly (*p* < 0.05) higher total ash levels than collectors, factories and retailers in the study regions. The samples obtained from Amhara region had the highest and lowest ash content. Raw milk channeled from farmers to collectors in the Oromia, SNNP, and Amhara regions resulted in variation of 4.92, 8.82, and 9.59%, respectively. There were also further ash value reductions in the above regions when they reached milk factories (5.17, 3.23, and 21.1%, respectively).

In SNNP and Amhara regions, the Ca content of raw milk collected from farmers were much higher than that of collectors. The Ca content in the value chain differs significantly (*p* < 0.05). In the current study, the amount of P in the Amhara region value chain differed considerably (*p* < 0.05). Samples collected from the Amhara region had the lowest and greatest levels of P. The P level was found to be between 62.7 and 84.2 mg/100g. The pH values ranged from 6.42 to 7.18 in all areas.

### 3.2 Adulterants in raw and pasteurized milk along value chain in the regions

The adulterants in raw and pasteurized milk along the value chains are shown in Table 3. Raw milk was found to be adulterated by water in all regions along value chain, with the exception of farmers in the Amhara region. In the Oromia region, we found no significant (*p* > 0.05) changes in the extent of water added percentage along the value chain. However, the extent of water adulteration was notably different in the Amhara region, followed by the SNNP region at milk factories and retailors. Of 120 pasteurized and raw milk samples, 32.5% had no added water. In the Amhara region, the amount of added water in milk factories was much higher than in farmers' and collectors. In this work, formalin was detected in 4 samples of raw milk at producer level in the Oromia region. Besides, out of 16 composite samples of raw milk, 1 sample was positive for starch.

**Table 3:** Adulterants of raw and pasteurized milk along value chain.

Study region	Value chain	Product type	N	Added water (%)	Formalin		Starch	
					Detected (n)	Not detected (n)	Detected (n)	Not detected(n)
Oromia	Farmers	Raw milk	16	1.18 ± 1.68^ab^	4	12		
	Collectors	Raw milk	16	2.46 ± 3.30^abc^			1	15
	Factories	Pasteurized	16	3.40 ± 2.02^abc^				
	Retailers	Pasteurized	16	2.32 ± 1.45^abc^				
	**Total/Grand mean**		**64**	**2.34** ± **2.11**	**4**	**12**	**1**	**15**
SNNP	Farmers	Raw milk	8	4.90 ± 5.58^bcd^				
	Collectors	Raw milk	8	5.57 ± 6.79^cd^				
	Factories	Pasteurized	8	8.60 ± 5.10^df^				
	Retailers	Pasteurized	8	12.2 ± 3.78^e^				
	**Total/Grand mean**		**32**	**7.82** ± **5.31**				
Amhara	Farmers	Raw milk	8	0.00 ± 0.00^a^				
	Collectors	Raw milk	8	5.0 ± 10.3^bcd^				
	Factories	Pasteurized	8	19.24 ± 8.91^f^				
	Retailers	Pasteurized	8	24.8 ± 6.86^g^				
	**Total/Grand mean**		**32**	**12.3** ± **6.53**				

All values were duplicate analysis (n = 2); mean values that do not share common letters, are different for p < 0.05.

### 3.3 Nutritional composition of cottage cheese along value chain in the regions

The nutritional quality of traditional cottage cheese along the value chain is presented in Table 4. In all regions, there was no significant (*p* > 0.05) difference in moisture, protein, fat, total ash, calcium, and phosphorus content between farmers and retailers. In the study regions, the mean moisture content of cheese samples ranged from 77.2 to 77.8%. 16.3–17.9% protein content was obtained. Fat content varied from 1.33 to 1.50% across all value chains in all regions. In Oromia, SNNP, and Amhara regions, the average Ca content was 37.8, 38.5, and 39.5 mg/100 g, respectively, whereas the average P content was 86.9, 87.7, and 90.2 mg/100 g. In present study, the pH value of Ethiopian cottage cheese had a lower pH range, between 3.63 and 3.80, compared to other soft cheese prepared in other country.

**Table 4:** Nutritional composition of cheese along value chain in three regions of Ethiopia.

Region	Value chain	N	Moisture (%)	Protein (%)	Fat (%)	Total ash (%)	Ca (mg/100 g)	P (mg/100 g)	Ph
Oromia	Farmers	8	76.2 ± 0.52	16.8 ± 2.22	1.33 ± 0.06	1.17 ± 0.07	37.8 ± 3.21	88.1 ± 9.74	3.80 ± 0.23
	Retailors	8	76.8 ± 1.12	16.3 ± 2.99	1.43 ± 0.13	1.16 ± 0.06	37.3 ± 3.53	85.7 ± 6.65	3.78 ± 0.33
	**Total/Grand mean**	**16**	**76.5**	**16.5**	**1.38**	**1.17**	**37.6**	**86.9**	**3.69**
SNNP	Farmers	4	77.4 ± 1.02	17.0 ± 4.08	1.36 ± 0.13	1.21 ± 0.07	36.6 ± 2.68	89.1 ± 13.7	3.69 ± 0.19
	Retailors	4	77.6 ± 0.87	16.8 ± 2.50	1.47 ± 0.14	1.18 ± 0.10	36.2 ± 5.04	86.3 ± 6.08	3.63 ± 0.16
	**Total/Grand mean**	**8**	**77.5**	**16.9**	**1.42**	**1.19**	**36.4**	**87.7**	**3.66**
Amhara	Farmers	4	77.1 ± 0.76	17.6 ± 3.52	1.44 ± 0.20	1.20 ± 0.04	40.1 ± 4.23	91 ± 7.84	3.77 ± 0.25
	Retailors	4	77.8 ± 0.75	17.9 ± 3.03	1.50 ± 0.21	1.14 ± 0.06	38.9 ± 2.62	89.5 ± 13.2	3.70 ± 0.26
	**Total/Grand mean**	**8**	**77.4**	**17.8**	**1.47**	**1.17**	**39.5**	**90.2**	**3.74**

All values were duplicate analysis (n = 2).

### 3.4 Comparison of raw and pasteurized milk with CES values

CES are obligatory requirements for essential quality characteristics of milk products that are needed to ensure the usefulness of a product and are incorporated into technical regulations of the country. Table 5 shows the comparison of fat, protein, and SNF of raw milk and pasteurized milk CES minimum requirement values. The results show that 17.2, 26.6, and 35.9% of raw milk samples met CES for fat, protein, and SNF, respectively. Of pasteurized samples analyzed, SNF (3.13%), protein (9.38%), and fat (26.6%) were found to fulfill the minimum requirements of CES. In the present work, the overall prevalence (fat, protein, and SNF) was 19.8%, which met the CES minimum requirement, whereas 80.2% did not.

**Table 5:** Comparison of proximate value of liquid milk with CES limit values.

Parameter	Product type	N	Mean values (%)	CES	Above CES (n)	Below CES (n)	Above CES (%)	Below CES (%)
Fat	Raw	64	2.74 ± 0.78^a^	Min 3.34	11	53	17.2	82.8
	Pasteurized	64	2.52 ± 0.54^b^	2.7–3.3	17	47	26.6	73.4
Protein	Raw	64	2.70 ± 0.89^a^	Min 3.07	17	47	26.6	73.4
	Pasteurized	64	2.41 ± 0.52^b^	Min 3.07	6	58	9.4	90.6
SNF	Raw	64	7.34 ± 1.62^a^	Min 8.33	23	41	35.9	64.1
	Pasteurized	64	6.44 ± 0.91^b^	Min 8.38	2	62	3.13	96.9
Total prevalence							19.8	80.2

All values were duplicate analysis (n = 2); mean values that do not share common letters, are different for p < 0.05; SNF=Solids Not Fat; CES = Compulsory Ethiopian Standard.

## 4 Discussion

### 4.1 Nutritional composition of raw and pasteurized milk along value chain in the regions

In the present work, we observed variability in the nutritional quality of milk products along value chain. In milk composition variability observed within the study. Total solids of cow's milk had differences along the value chain. This disparity must be influenced by breeds, stage of lactation, season, feeding, age, health, and physiological status of the animal [27,28]. Feeding patterns, geographical locations, different analytical methods used, and sample size can also be factors in milk composition variability [13]. The study done by Ref. [28] found 12.8% the mean total solids in raw milk in dairy farms with min and max values of 9.30% and 23.9%. Bovine milk has a total solids content of 13–15% [29].

In Ethiopia, urban and peri-urban production systems use exotic cow breeds (Jersey and Holstein-Friesian) and crossbreds on smallholder and commercial farms for milk production [30,31] which could cause variability in the nutritional composition of raw milk. The total milk contents at origin (raw milk) influence the posterior steps of the chain and they are several farmers sources with different composition. Ref [13] stated that breed, season, and the interaction between composition elements of camel milk found that fat and total solids were significant moderators of protein; moreover, fat was a significant moderator of total solid content. In this work, the variations in crude protein content of milk in farmers could also be the use of different types of concentrate feed, green forages, and crop residue accessible in the study regions. The main feed sources in the highlands of Ethiopia (small and medium-sized dairy farms) are agro-industrial byproducts and purchased roughage [31,32]. The study by Ref. [33] observed a positive correlation between supplementation of corn grain and milk protein concentration. Moreover [34], reported a significant increase in percentage milk protein when cows are fed fresh grass and varying ratios of soybean and cereal concentrate. The typical protein content of bovine milk ranged from 2.50 to 3.50% [29].

In present study, a decreased mean fat value was achieved, implying that some creams were skimmed from raw milk during pasteurization, though multiple factors influenced fat content (Table 3). Milk factories have a practice of skimming the raw milk to standardize fat content. Fat-soluble vitamins may be lost, affecting daily dietary requirements for milk consumers, particularly children. In Addis Ababa city, mean 3.30% fat concentration were found in pasteurized and raw milk collected from retail shops (n = 103) [19] which were higher result than current values. Bovine milk has 3.80–5.50% of fat values [29]. Cow milk fat yield increased with grass feeding and decreased with concentrate feeding [35].

The reduction in lactose content in some milk factories in the Amhara region could be due to water adulteration practices. Water adulteration is also indicated by a lower concentration of lactose [36]. In contrast to milk fat and protein content, concentrations of lactose are relatively constant and are not subject to significant variation outside of lactational changes [35,37,38]. In Bangladesh, the research done by Ref. [39] found lactose content for milkmen (4.17%), dairy farms (4.23%) and pasteurized milk (4.3%), which were higher than current findings. In different studies, low lactose value could be caused by sick cows (udder inflammation), which can alter the composition, properties, and behavior of milk. In Ethiopia, mastitis is a serious problem at farmer's level [40]. Ref [35] observed significantly higher lactose yield but not lactose percentage in perennial ryegrass/white clover pasture and mixed ration than in perennial ryegrass. Ref [19] found that there was 4.30% lactose value in Addis Ababa retail shops selling raw and pasteurized milk. Lactose was determined using Lactoscan. Ref [36] obtained 3.89% mean of lactose concentration in raw milk (n = 30) and HTST pasteurized milk (n = 30) collected from farmers supplying to milk factories and retailers with minimum (2.12%) and maximum (5.11%) values.

Total ash content is an index of total mineral matter in liquid milk. Lower raw milk quality routed from various milk unions and water dilution practices by concentration (Table 5) could be the cause of the decreases in total ash values. In Tanzania, a total of 98 raw cow milk samples from selected smallholder dairy farms were analyzed and found to have a mean ash content of 0.70% (range 0.40–1.81%) [28], which were greater values than present finding. In India, research conducted by Ref. [41] at a dairy research farm of Kankrej breed milk observed 0.76% ash. Total ash content (0.68–0.78%) was obtained in raw milk, pasteurized milk, and ultra-high temperature (UHT) milk collected from the Bangladesh retail market [18].

In this study, when raw milk at collectors’ level (before pasteurization) and after pasteurization were compared, significant reductions in major nutritional components (protein and lactose) were observed. Pasteurization did not have a negative effect on the lactose and protein content of pasteurized milk, which is similar to raw milk [42]. The nutrient reductions could be attributed to variations in the mixing of different nutrient levels in the dairy value chain (various farmers, collectors, and milk factories) prior to pasteurization. In Ethiopia, 95% of milk is channeled through the informal market [8]. Generally, milk composition is influenced by breed, age, level of parity, health status, stage of lactation, diet; the intensity of management; milking interval; number of viable pregnancies; processing procedures after milk collection; and ambient environmental temperature and seasonality [11,12,29,43].

In this study, lower levels of Ca and P were observed, which could be due to lower protein content. The majority of Ca and P in milk is incorporated into the casein micelle as colloidal calcium phosphate [38]. The grass feeding system produced milk with significantly higher Ca and P levels than both the perennial ryegrass/white clover pasture and total mixed ration systems [38,44]. The study done by Ref. [45] found a 74.9–78.4 mg/100 g average content of Ca in the milk of Holstein Friesian cows between three lactation stages and concluded that lactation stage had a significant effect on Ca concentration. In India [41], reported 120.2 mg/100 g Ca and 88.1 mg/100 g P contents from the Kankre*j* breed. According to Ref. [46], the present findings were lower than the Ca range (91–120 mg/100g) and P (84–95 mg/100 g). The wide range in particular mineral concentration (Ca and P) in cow's milk may be attributed to feeding system, lactation stage, environmental, and genetic factors [15,47].

The pH values of milk from milk collectors were slightly acidic compared to normal pH value (6.6–6.8). This could be due to the long distance to collection centers/unions, which provides an opportunity for lactic acid fermentation by consuming lactose to reduce pH to an acidic condition. However, a slight decrease in acidity was recorded at milk factories and retailers' level in the Amhara region. This increased pH could be due to the pasteurization effect together with extraneous water adulterations on lowering whey protein associating with the micelles and minerals imbalance. The research conducted by Ref. [28] reported that out of 98 raw milk test samples, 35 samples had 6.6 pH values, 60 samples were in the normal range (6.6–6.8) and 3 samples were >6.8.

### 4.2 Adulterants in raw and pasteurized milk along value chain in the regions

Raw milk is adulterated with water in some countries to increase its volume for economic reasons. It also preserved with formalin to extend its shelf life for long-distance transportation to reach at milk collection centers or processing factories. In this study, the higher extent of water adulteration in milk factories than producer and collectors could be due to residual water in milk handling and storage containers as a result of value chain poor cleaning and drying practices. Ref [36,48] stated that the operation system in old factories could be a source for water addition by technical faults. In present work, water adulteration practices might be intentionally for gaining income. For accuracy measurement of added water in milk, cryoscope would be a better method than lactoscan. Ref [19] reported 61.2% had extent of added water in fresh raw milk (n = 50) and branded pasteurized milk (n = 53) collected from retail market in Addis Ababa city which is lower than this study results. They also found positive association between extent of added water in raw milk and lower price. The study by Ref. [36] in South Africa found high extent of added water percentage in pasteurized milk from milk factories than raw bulk milk, with mean 21 ± 14.6%. In Nepal, table sugar was detected in raw milk (9 samples) and pasteurized milk (18 samples), out of 20 samples tested. However, formalin, starch and neutralizer were not detected [49]. Similar study by Ref. [39] conducted to evaluate qualitative detection of adulterants in raw and pasteurized milk from local market (n = 20) and found 70% samples adulterated with neutralizers where as 30% were adulterated with skimmed milk powder. However, detergent and starch were not positive.

Adulterating milk using untreated low-quality water and adding formalin to it poses a public health concern to dairy consumers. Formalin is a highly poisonous chemical that destroys the liver and kidneys and is linked to cancer. In this work, the extent of water adulteration is a critical adulterant and it is a main reason for lower nutritional quality of raw and pasteurized milk. This imply that milk consumers are taking less nutrients and are paying a price for liquid milk that is substandard.

### 4.3 Nutritional composition of cottage cheese along value chain in the regions

In this work, no variation in cottage nutritional quality was observed. This suggests that farmers used similar processing and storage procedures while making traditional cheese. Ref [20] assessed the nutritional quality of cottage cheese from the Amhara region found that moisture content was high (78.7–78.5%). These values were slightly higher than those observed in the existing research. Cheese with a higher moisture content is more susceptible to quality and safety changes, resulting in a shorter shelf life that necessitates proper storage conditions. The moisture content of soft Mexican cheese (Aro cheese) made with pasteurized whole milk or milk with 2% fat, mesophilic lactic acid bacteria (0.25%), and chymosin (0.02%) with mild agitation was 59.4–63.7% [50] which was lower than the study results. In Egypt, cheese types prepared from cow milk (pH 6.55–6.85), pasteurized at 72 °C/15–20 s, 0.01% calcium chloride, 10% mesophilic starter culture (Lactococcus lactis subsp. Lactis and Lactococcus lactissubsp. cremoris) and 0.5% rennet had moisture 82.2% for free fat cottage cheese and 79.7% reduced fat cottage cheese [51] which were higher results than current finding. The ingredients used in cheese production as well as the processing methods affects moisture content of cottage cheese.

In present research, lower fat values were obtained, due to the removal of cream during traditional cheese processing [20], finding (1.42–1.44%) were consistent with current results, slight differences could be due to fat extraction method analysis. Higher protein percentage was found which were slightly higher than the report by Ref. [20]. Cottage cheese is a great source of protein for children and people who are trying to lose weight. Differences in cheese protein content could be raw milk quality used, fermentation duration, and processing procedures. Ref [51] evaluated proximate composition of different cheese types for cheesecake preparation and they found 13.9% protein and 0.41% fat for free fat cottage cheese whereas for reduced fat-cottage cheese were 12.9% protein and 3.14% fat. They also reported total ash for cream cheese, full fat cheese, reduced fat cheese and free fat cheese were 1.33, 1.49, 1.62 and 1.78%, respectively.

Cottage cheese is a good source of minerals like Ca and P, and it is an important food in the diet of both young and old people [51]. The results for Ca and P were lower than the finding by Ref. [20] who reported Ca (40.5 mg/100 g) and P (126.1 mg/100 g) on average. Lower Ca and P contents were obtained might be due to lower amount in original raw milk used for cheese making in the area.

Ethiopian cheese had high acidity compared to soft cheese producer in other country. The main reason is that cheese preparation and processing methods. Higher numbers of lactic acid bacteria are produced during ergo fermentation to make cheese, which involves reducing pH levels. Moreover, there may also changes in pH values during cheese storage practices by different farmers and retailers.

### 4.4 Comparison of raw and pasteurized milk with CES values

Comparison of liquid milk with CES indicate pasteurized milk samples met CES higher than raw milk may be due to standardization (removal of fat) of raw milk during pasteurization process (Table 5). In present work, 80.2% the overall prevalence (fat, protein and SNF) did not meet CES minimum requirement. In Kenya, the majority of the milk tested met Kenya Bureau of Standards (KeBS) for fat and SNF. However, protein was below the standard in farmers and informal milk collection, except at formal bulking [15] which are lower than current results. Raw milk and branded pasteurized milk were in conformity with Bangladesh Standard and Testing Institution (BSTI). However fat content of pasteurized milk was not fulfilled the requirement which was not less than 3.40% [18].

## 5 Conclusions

In this study, it can be concluded that the overall 80.2% for fat, protein, and SNF in raw and pasteurized milk was below CES minimum requirement. Moreover, raw and pasteurized milk had poor nutritional content and varied along the value chain. Furthermore, there is milk fraud where all value chains add water into liquid milk and people are consuming fewer nutrients and paying for milk that is substandard. However, Ethiopian cottage cheese had good nutritional quality of protein content. Therefore, regulatory and inspection bodies should strictly monitor the quality of dairy products from production to consumption. Besides, quantification of formalin and other adulterants in milk products need to be further studied. Furthermore, training and awareness creation should be provided to all value chains to improve the quality of milk products, protect public health and fulfil nutritional requirements.

## Author contribution statement

Haftom Zebib Abraha, Dawit Abate, Ashagrie Zewdu Woldegiorgis: Conceived and designed the experiments; Performed the experiments; Analyzed and interpreted the data; Contributed reagents, materials, analysis tools or data; Wrote the paper.

## Data availability statement

Data included in article/supplementary material/referenced in article.

## Funding statement

This work was supported by the 10.13039/100000865Bill & Melinda Gates Foundation and The 10.13039/501100020171Foreign, Commonwealth and Development Office of UK (Grant Number of INV-008459).

## Prevention of covid 19 during sampling and laboratory analysis

To prevent COVID-19 during sample collection and laboratory analysis, we followed the ENSURE E-dairy project prevention and control guidelines adapted from WHO and the Ministry of Health of Ethiopia, which required participants to maintain social distance, wear a face mask, and use hand sanitizers. Moreover, the project had also arranged enough cars to keep a social distance during fieldwork traveling between researchers to guarantee the wellbeing of project participants.

## Declaration of competing interest

The authors declare that they have no known competing financial interests or personal relationships that could have appeared to influence the work reported in this paper.
